# The impact of mode of delivery on parent-infant-bonding and the mediating role of birth experience: a comparison of mothers and fathers within the longitudinal cohort study DREAM

**DOI:** 10.1186/s12884-023-05611-8

**Published:** 2023-04-25

**Authors:** Svenja Döblin, Lara Seefeld, Victoria Weise, Marie Kopp, Susanne Knappe, Eva Asselmann, Julia Martini, Susan Garthus-Niegel

**Affiliations:** 1 Institute and Policlinic of Occupational and Social Medicine, Faculty of Medicine, Technische Universität Dresden, Dresden, Germany; 2 Department of Psychotherapy and Psychosomatic Medicine, Faculty of Medicine TU Dresden, Dresden, Germany; 3 Evangelische Hochschule Dresden (Ehs), University of Applied Sciences for Social Work, Education and Nursing, Dresden, Germany; 4 Faculty of Health, HMU Health and Medical University, Potsdam, Germany; 5 Institute of Clinical Psychology and Psychotherapy, Technische Universität Dresden, Dresden, Germany; 6 Department of Psychiatry & Psychotherapy, Faculty of Medicine, Carl Gustav Carus University Hospital, Technische Universität Dresden, Dresden, Germany; 7 Institute for Systems Medicine (ISM), Faculty of Medicine, Medical School Hamburg, Hamburg, Germany; 8 Department of Childhood and Families, Norwegian Institute of Public Health, Oslo, Norway; 9 Institute and Outpatient Clinics of Occupational and Social Medicine, Faculty of Medicine of the Technische Universität Dresden, Fetscherstraße 74, 01307 Dresden, Germany

**Keywords:** Mode of delivery, Cesarean section, Birth experience, Mother-infant-bonding, Father-infant-bonding, Moderated mediation analysis, DREAM study

## Abstract

**Background:**

The association between mode of delivery (MOD) and parent-infant-bonding has only been studied in mothers and findings have been inconclusive. The aim of this study was to prospectively investigate how MOD relates to postpartum parent-infant-bonding in both mothers and fathers and whether these associations are mediated by birth experience.

**Methods:**

This study is part of the prospective cohort study “Dresden Study on Parenting, Work, and Mental Health” (DREAM). Our sample comprised *N* = 1,780 participants who completed quantitative questionnaires during pregnancy as well as 8 weeks and 14 months postpartum. MOD was dummy coded, contrasting spontaneous vaginal delivery against vaginal delivery induced by drugs, operative vaginal delivery, planned, and unplanned cesarean section. Parent-infant bonding and birth experience were assessed using validated scales. A moderated mediation analysis based on ordinary least square (OLS) regression and bootstrapped estimates was conducted, considering relevant confounding variables.

**Results:**

Compared to spontaneous vaginal delivery, all categories of MOD predicted more negative birth experiences in both parents. A more positive birth experience predicted stronger parent-infant-bonding at 8 weeks, but not at 14 months postpartum. Mothers who delivered via cesarean section (planned or unplanned) reported stronger parent-infant-bonding at 8 weeks and 14 months postpartum. In fathers, only unplanned cesarean section was associated with stronger parent-infant-bonding at 8 weeks postpartum. At 8 weeks postpartum, birth experience mediated the association between a vaginal delivery induced by drugs and a planned cesarean section and mother-infant-bonding and between a vaginal delivery induced by drugs, an operative vaginal delivery, and planned cesarean section and father-infant-bonding. At 14 months postpartum, birth experience mediated the association between a vaginal delivery induced by drugs, operative vaginal delivery, and planned cesarean section and parent-infant-bonding in both parents.

**Conclusions:**

The results emphasize the importance of the birth experience for parent-infant-bonding in both mothers and fathers. Further research should address the mechanisms by which parents with an unplanned cesarean section establish stronger parent-infant-bonding compared to parents whose baby was delivered via spontaneous vaginal delivery, despite their overall more negative birth experiences.

## Background

Childbirth is considered as one of the most powerful life experiences, both physically and psychologically, leaving an impression for everyone involved in this process [1–3]. With increasing cesarean section (CS) rates in industrialized countries over the past decades [4, 5], approximately 30% of infants in Germany are delivered via CS [5, 6]. However, in Dresden, where the present study is based, the number is lower with 19.5% of women giving birth by CS [7]. An unplanned CS and instrumental vaginal delivery have been linked to more negative birth experiences [8–14] and an increased risk of postpartum mental health problems in mothers [15]. Most women wish for a natural birth without obstetric interventions, as well as a positive birth experience [16]. Bossano et al. [17] showed that the mother’s perception of the birth experience persists even a decade after delivery, which again highlights the significance of this experience as a major life event. Mothers who have had a negative subjective birth experience seem to report poorer mother-infant-bonding [18], although the literature is sparse and often focuses on caregiving [19]. Still, facilitating a positive birth experience might enable stronger mother-infant-bonding. Bicking Kinsey et al. [20] emphasize the often occurring, problematic synonymous use of the terms mother-infant-bonding and attachment in this research field. Based on the authors’ given definition, parent-infant-bonding is to be understood as the emotional connectedness that parents experience with their infant [20]. How parents’ bond with their infants and the attachment infants feel to them have an impact on several relevant outcomes. Indeed, weaker parent-infant-bonding is associated with greater parenting stress in mothers and fathers [21], which is in turn negatively associated with parents’ responsiveness to the infant’s needs [22]. Greater parental responsiveness on the other hand seems to be linked to greater attachment security of the infant [23]. This is especially important because secure attachment of the infant is associated with positive developmental and health-related outcomes of the child [24]. Thus, parent-infant-bonding is a concept of great importance that furthermore seems to be characterized by moderate to high stability, measured at 6 and 24 months postpartum [25]. Investigating the mode of delivery (MOD) and birth experience as potential predictors could therefore identify possible targets to influence the establishment and strengthening of this long-lasting bond.

However, it remains unclear how MOD and parent-infant-bonding are linked to each other. There is limited, mixed evidence regarding this association focussing on mothers. Some studies indicate little to no difference in mother-infant-bonding, regardless of whether the mother undergoes a CS (planned or unplanned) or a vaginal delivery [26–29]. Then again, there are some findings suggesting a significant association between MOD and mother-infant-bonding. For instance, a prospective study contrasting CS and vaginal delivery showed that women who had an unplanned CS reported weaker bonding [30]. Similarly, Ishii et al. [31] found that women who had a vaginal delivery reported stronger mother-infant-bonding than women who underwent a planned CS. Also, a qualitative study interviewing women who had an unplanned CS reported an interruption of the initial bond between mother and infant and a feeling of disconnectedness due to the separation after childbirth [32]. In fact, only 61.9% of women who underwent a CS (planned or unplanned) held their babies for the first time within the first hour after childbirth, compared to 98.4% of women who had a vaginal delivery [33]. Brubaker et al. [34] showed that seeing, holding, and feeding the baby right after childbirth is associated with a more positive birth experience, both of which were more likely for mothers with a vaginal delivery than a CS. Thus, women who underwent a CS reported less overall positive birth experiences. However, if the first contact between mother and child occurred within five minutes or less after delivery along with first feeding within 30 min after delivery, mothers with a CS (planned and unplanned) reported in fact a more positive birth experience than mothers giving birth via vaginal delivery, at the same time of first encounter [34]. Therefore, this seems to be an important aspect to consider in the evaluation of the impact of MOD on the birth experience, as there might be varying customary practices after a CS across different hospitals. The Baby-friendly Hospital Initiative (BFHI), a global effort developed by the World Health Organization (WHO) and United Nations Children’s Fund (UNICEF), states that undisturbed skin-to-skin contact is also recommended right after a CS, as long as the mother is alert and able to hold the baby [35]. Although this practice has numerous positive health-related outcomes [36], it is rarely provided after a CS in standard hospital obstetric cares [37–39]. Still, as two hospitals in Dresden are certified as BFH, it is conceivable that the number of mothers holding their baby immediately or early after CS could be higher in the present study than the 61.9% found by Chalmers et al. [33].

As briefly indicated above, previous research focused mainly on mothers [40, 41]. However, the greater inclusion of fathers in this research is critical. Most men attend the birth of their child these days [42] and describe the experience as positive [43, 44]. Indeed, it seems to be the moment of childbirth that men point out as the transitioning moment to fatherhood [45] and involving the father in this can strengthen the family [46]. This is also in line with motivational aspects in attending childbirth for fathers. By being present during birth, most fathers want to engage in this unique experience and provide support for their partner [47, 48]. Cutting the umbilical cord is associated with an increase of father-infant-bonding [49]. Sound evidence regarding the link between MOD and the birth experience in fathers is however sparse and inconclusive. Examining an unplanned CS and instrumental vaginal delivery, Johansson et al. [44] found an association for both MODs with a more negative paternal birth experience, while Nystedt et al. [10] found such association only for an unplanned CS. Besides, Hildingsson et al. [50] studied instrumental vaginal deliveries and found them to be associated with a more negative paternal birth experience. Still, men seem to report a negative birth experience less often compared to women [51]. In addition, Kress et al. [52] found that an unplanned CS predicted birth-related PTSD symptoms in mothers but not in fathers. Hence, there might be differential effects regarding the impact of the MOD on the birth experience in mothers and fathers. However, this needs further clarification. Also, to the best of our knowledge, the link between MOD and father-infant-bonding has not been studied yet. Addressing this research gap will be a novelty of the current study.

**Fig. 1:**
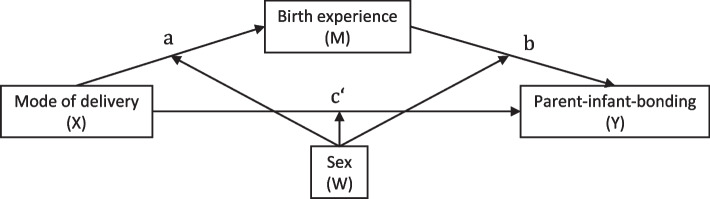
Conceptual model of the proposed moderated mediation analysis

Therefore, the aim of this study is to examine the impact of MOD on parent-infant-bonding, at 8 weeks and 14 months postpartum, including both mothers and fathers. Additionally, it will be investigated whether this association is mediated by birth experience. Since we are particularly interested in comparing mothers and fathers, parental sex is included as a moderator, resulting in a moderated mediation model [53] for the analyses, as illustrated in Fig. 1.

## Methods

### Design

The present study is part of the Dresden Study on Parenting, Work, and Mental Health (“DResdner Studie zu Elternschaft, Arbeit und Mentaler Gesundheit”; DREAM). This prospective cohort study investigates the association between parental work participation and distribution, stress factors and their impact on various perinatal outcomes as well as the long-term mental and somatic health of the family. Participants are (expectant) parents, recruited at information evenings at hospitals and birth preparation courses in Dresden, Germany, and surroundings. At present, the longitudinal design consists of six measurement points: T1 during pregnancy, T2 at 8 weeks after the expected birth date, T3 at 14 months after the actual birth (the date the child was born), T4 at 2 years, T5 at 3 years, and T6 at 4.5 years postpartum. For this study, data of the first three measurement points (T1─T3) were used. Participants completed quantitative questionnaires at each measurement point, either in paper and pencil format or online. Study participation is voluntary and not financially compensated. However, small incentives (e.g., rompers or small books) are provided with every follow-up questionnaire. All participants provided written informed consent. Kress et al. [54] provide further information on the DREAM study design.

### Sample

By December^3rd^ 2020, the day of data extraction, *n* = 3,827 mothers and fathers were included in the cohort. Inclusion criteria for the present study were expecting one child (singleton pregnancy) and paternal presence during childbirth. Also, the questionnaires at T1, T2, and T3 had to be completed in a defined time frame for this study. Particularly, parents were excluded if T1 was completed after childbirth because the confounding variable prenatal symptoms of depression required the measurement during pregnancy. Further, parents were excluded if T2 was completed earlier than 6 weeks or later than 16 weeks postpartum, and/or if T3 was completed earlier than 12 months or later than 16 months postpartum. This is substantial, because the personal assessment of birth experience and parent-infant-bonding may be time-dependent [55, 56] and, consequently, may change over time. Our selection of time frames is consistent with Seefeld et al. [18]. Retention rates of this sample can be provided upon request. Applying these criteria, the final sample comprised *n* = 1,780 participants.

### Measures

#### Predictors and outcome

Information about mode of delivery (MOD) was retrieved from maternal report based on maternity records [57] at T2. Categories were spontaneous vaginal delivery (spontaneous VD), vaginal delivery induced by drugs (induced VD), vaginal operative birth (with forceps or vacuum extraction; operative VD), planned cesarean section due to personal reasons or planned caesarean section due to medical reasons (these were combined into planned CS prior to analyses), and unplanned cesarean section (unplanned CS). For data analysis, MOD was dummy coded (with spontaneous VD as reference category).

Birth experience was assessed using the German version of the Salmon’s Item List (SIL) in mothers and fathers at T2 [58]. The SIL contains 20 items on four scales (fulfilment, emotional adaption, physical discomfort, negative emotional experience). Each item is divided in its positive and negative valence, presented as an anchor word (e.g., item 2: fulfilled vs. not fulfilled). The participants were asked to rate each item on a Likert scale ranging from 1 to 7. The total score is the sum of all items, ranging from 0 to 120. Low scores indicate a more negative birth experience. The reliability of the SIL was excellent (Cronbach’s α = 0.91).

Parent-infant-bonding was measured by the German version of the Postpartum Bonding Questionnaire (PBQ) [59–61] at T2 and T3 in mothers and fathers. The PBQ is a screening instrument for bonding disorders. It consists of 25 items capturing four subscales: impaired bonding, rejection and anger, anxiety about care, and risk of abuse. Participants were asked to think of the most difficult time they experienced with their child and then rate how often the stated situations occurred, with answers ranging from “never” to “always” on a 6-point Likert scale. The sum scores yield scores for each subscale and a total score, ranging from 0 to 125. Low scores indicate strong bonding. The statistical analyses were based on the total score. The reliability of the PBQ was good at both T2 (Cronbach’s α = 0.87) and T3 (Cronbach’s α = 0.89).

#### Confounding variables

The selection of potential confounding variables was based on the existing literature [12, 14, 25, 28, 29, 56, 62–73]. In previous research, the following variables have been shown to be associated with birth experience or parent-infant-bonding: parity, academic degree, prenatal symptoms of depression, birth complications, and the timing of holding the baby for the first time after childbirth. Parity was assessed at T1. Similarly, academic degree was measured at T1, which was then dichotomised into holding a university degree (bachelor’s degree or higher) or not. Prenatal symptoms of depression were assessed at T1 using the German version of the Edinburgh Postnatal Depression Scale (EPDS) [74–76]. The EPDS consists of 10 items capturing symptoms of depression during the last week, and each item scores on a scale from 0 to 3, yielding a total score potentially ranging from 0 to 30. The reliability of the EPDS was good (Cronbach’s α = 0.84). Birth complications, e.g., heavy bleeding or perineal tears, were assessed at T2 using information derived from maternity records [57]. An index was created indicating their frequency (0,1,2, and ≥ 3).

Mothers and fathers were asked at T2 about when they held their baby for the first time, i.e., within the first minutes, within the first hour, or more than an hour after childbirth.

All variables were assessed in both mothers and fathers, except for MOD and birth complications. These were based on maternal report and then assigned to both partners prior to analyses.

### Data analysis

Statistical analyses were conducted with IBM SPSS Statistics Version 27.0 [77] and the SPSS modelling tool PROCESS [78]. In case of missing values in the psychometric scales SIL, PBQ, or EPDS the participant’s mean value was used if no more than 20% of the items were missing. Data were checked for outliers with case-by-case diagnosis and standardized residuals. Their impact on the regression estimates was diagnosed with Cook’s Distance, centred leverage values, DFBETAS (i.e., differences in beta coefficients), and standardized DFBETAS. No cases needed to be excluded in the analyses. A moderated mediation analysis [53] based on ordinary least square (OLS) regression was conducted. Bootstrapping with 5,000 iterations was computed. As parent-infant-bonding was assessed at T2 and T3, two analyses were carried out. A level of significance of *p* < 0.05 with 95% confidence intervals was applied. Unstandardized regression coefficients (*b*) and HC4 heteroskedasticity-consistent standard errors [79] are reported. The presentation of regression equations and results is based on recommendations of Hayes [78, 80], Hayes et al. [81], and Preacher et al. [53].

**Fig. 2:**
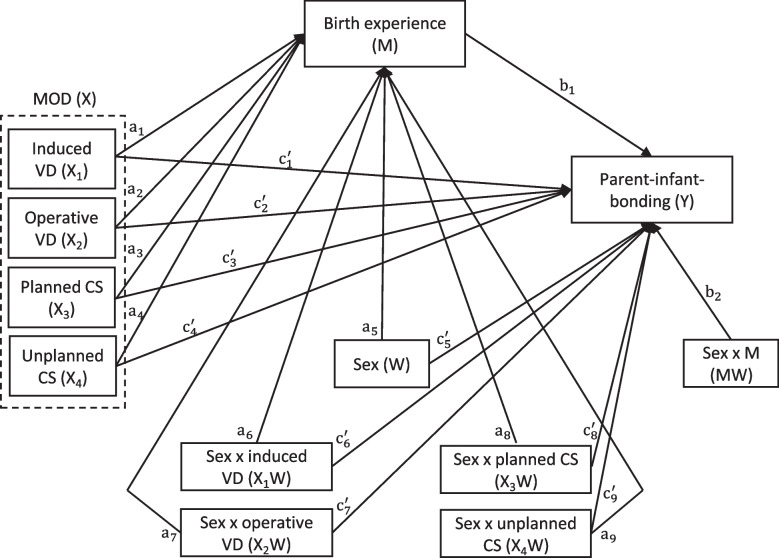
Statistical model of the proposed moderated mediation analysis. *Note.* MOD = mode of delivery; VD = vaginal delivery; CS = caesarean section

The hypothesised conceptual model in Fig. 1 is converted into a statistical model of the moderated mediation analysis which is shown in Fig. 2.

For a detailed discussion of the advantages of a moderated mediation analysis over, e.g., subgroup analysis or multi-group structural equation modelling, see Hayes [78]. Each effect in our model can be conditional on the values of the moderator (i.e., differ between mothers and fathers), entailing implications that are important for both the presentation and interpretation of results. As parental sex is a dichotomous variable in this study, the pick-a-point approach [82] was applied, estimating the conditional effects [53] at value 1 (mothers) and value 2 (fathers) of the moderator in all analyses. With the predictor MOD being a categorical variable, contrasting four categories against the reference category in its influence on subsequent variables, the conditional effects of interest become *relative* conditional effects. Therefore, whenever the investigated path concerned the predictor, we analyzed and interpreted the relative conditional effects. To depict these effects, a notation with θ is used [53, 78]: a vector comprising the sum of regression coefficients for which the particular effect is calculated (e.g., ${\theta }_{VBD\to M}\left|W\right.$= ${a}_{1}{X}_{1}+{a}_{6}{X}_{1}W$). The underlying OLS regression coefficients are reported in Tables 4 and 5, but not interpreted. As parent-infant-bonding is assessed at T2 and T3, analysis 1 refers to the former and analysis 2 refers to the latter.

## Results

### Sample characteristics

The final sample consisted of *N* = 1,780 participants (mothers: *n* = 1,079; fathers: *n* = 701). The characteristics of the sample are displayed in Table 1. At recruitment (T1), the mean week of gestation was 30.23 (*SD* = 6.06), ranging from 9 to 41. Mean age at T1 was 30.13 years (*SD* = 3.94) for mothers and 32.23 years (*SD* = 4.89) for fathers. Most of the participating parents were born in Germany (mothers: 95.9%; fathers: 97.8%), living in a permanent relationship (mothers: *n* = 99.1%; fathers: *n* = 99.9%), and were expecting their first child (mothers: 79.6%; fathers: 79.4%). The CS rate in this study was 15% in mothers, which is approximately 5% lower than the number previously reported for Dresden [7]. The SIL’s mean score was 78.97 for mothers (*SD* = 20.25) and 93.10 for fathers (*SD* = 14.83). For mothers, the mean score of the PBQ was 12.85 (*SD* = 9.84) at T2 and 13.80 (*SD* = 10.04) at T3. For fathers, the mean score of the PBQ was 12.63 (*SD* = 8.22) at T2 and 13.17 (*SD* = 8.22) at T3. The intercorrelations between all study variables are displayed in Table 2.

**Table 1:** Sample characteristics

	Mothers		Fathers	
	*M* (*SD*)	Range	*M* (*SD*)	Range
Age in years^d^	30.13 (3.94)	15─43	32.23 (4.89)	21─56
Week of gestation^d^	30.23 (6.06)	9─41	-	-
SIL^e^	78.97 (20.25)	12─120	93.10 (14.83)	23.33─120
PBQ^e^	12.85 (9.84)	0─93	12.63 (8.22)	0─54
PBQ^f^	13.80 (10.04)	0─102	13.17 (8.22)	0─44
EPDS^d^	5.52 (4.13)	0─23	3.92 (3.72)	0─23
	*n* ^a^	*%* ^b^	*n* ^a^	*%* ^b^
Country of birth^d^				
Germany	1032	95.9	681	97.8
Other	44	4.1	15	2.2
Partnership status^d^				
Partner	1064	99.1	695	99.9
No partner	10	0.9	1	0.1
Academic degree^d^				
University degree	636	59.2	396	57.4
No university degree	439	40.8	294	42.6
Employment status^c,d^				
Full-time	487	45.1	582	83.0
Part-time	187	17.3	59	8.4
Parental leave	149	13.8	0	0
Unemployed	22	2.0	7	1.0
Parity^d^				
Primiparous	852	79.6	543	79.4
Multiparous	218	20.4	141	20.6
Mode of delivery^e^				
Spontaneous VD	635	59.8	392	60.3
Induced VD	186	17.5	113	17.4
Operative VD	82	7.7	53	8.2
Planned CS	72	6.8	41	6.3
Unplanned CS	87	8.2	51	7.8
Timing of holding the baby^e^				
Within first minutes	919	86.1	318	45.6
Within first hour	101	9.5	272	39.0
> 1 h after childbirth	47	4.4	107	15.4
Birth complications^e^				
0	577	53.5	361	54.4
1	363	33.6	218	32.8
2	118	11.0	72	10.8
≥ 3	21	1.9	13	2.0

*SIL* Salmon’s Item List, *PBQ* Postpartum Bonding Questionnaire, *T2* around 8 weeks after the expected birth date, *T3* around 14 months after the actual birth date, *EPDS* Edinburgh Postnatal Depression Scale, *VD* vaginal delivery, *CS* cesarean section

^a^*n* varies slightly across variables due to missing data of some participants

^b^Valid percentages are displayed

^c^Item allowed multiple choice, thus participants could report more than one employment status

^d^T1 during pregnancy

^e^T2 around 8 weeks after the expected birth date

^f^T3 around 14 months after the actual birth date

**Table 2:** Spearman correlations including all study variables

	1.	2.	3.	4.	5.	6.	7.	8.	9.	10.	11.	12.	13.	14.
1. Spontaneous VD^c^	-													
2. Induced VD^c^	^a^	-												
3. Operative VD^c^	^a^	^a^	-											
4. Planned CS^c^	^a^	^a^	^a^	-										
5. Unplanned CS^c^	^a^	^a^	^a^	^a^	-									
6. SIL^c^	.25^**^	-.06^*^	-.16^**^	-.02	-.19^**^	-								
7. PBQ^c^	-.01	-.02	.05^*^	-.02	.02	-.30^**^	-							
8. PBQ^d^	.02	-.01	-.00	-.02	-.00	-.23^**^	.64^**^	-						
9. Parental sex^b^	.01	-.00	.01	-.01	-.01	.36^**^	.02	-.01	-					
10. Parity^b^	.07^**^	-.01	-.11^**^	.02	-.03	.15^**^	-.11^**^	-.02	.00	-				
11. Birth complications^c^	.04	.08^**^	.02	-.14^**^	-.07^**^	-.08^**^	.05^*^	.04	-.01	-.13^**^	-			
12. Timing of holding the baby^c^	-.16^**^	-.04	.02	.10^**^	.22^**^	.05	.05	.03	.42^**^	-.03	-.05	-		
13. EPDS^b^	-.02	.04	-.01	.02	-.03	-.18^**^	.20^**^	.22^**^	-.22^**^	.10^**^	-.03	-.09^**^	-	
14. Academic degree^b^	.03	-.05^*^	.02	-.02	.02	-.08^**^	.15^**^	.13^**^	-.02	-.04	.01	-.00	-.09^**^	-

Two-tailed testing. *MOD* mode of delivery, *VD* vaginal delivery, *CS* caesarean section, *SIL* Salmon’s Item List, *PBQ* Postpartum Bonding Questionnaire, *EPDS* Edinburgh Postnatal Depression scale; *p < .05; **p < .01

^a^As the correlation coefficients would not indicate a meaningful association in terms of content they are not reported

^b^T1 during pregnancy

^c^T2 around 8 weeks after the expected birth date

^d^T3 around 14 months after the actual birth date

### Dropout analyses

Dropout analyses were carried out to examine whether completers and non-completers differed statistically significantly from each other regarding the predictors, confounding variables, and sociodemographic characteristics (tables provided upon request). Among mothers, non-completers reported higher mean EPDS scores (*M* = 6.69, *SD* = 4.72, *n* = 141) than completers (*M* = 5.51, *SD* = 4.13, *n* = 1041), *t*(1180) = 3.13, *p* = 0.002, *d* = 0.28. Additionally, non-completers more often had no university-degree (54.9% against 40.8%), χ^2^(1) = 10.20, *p* = 0.001, ϕ = 0.09 and were more often unemployed (4.8% against 2.0%), χ^2^(1) = 4.23, *p* = 0.040, ϕ = -0.06 than completers. Also, non-completers had a spontaneous VD less often (46.9% against 59.8%) and an unplanned CS more often (13.3% against 8.2%) compared to completers, χ^2^(4) = 10.44, *p* = 0.034, V = 0.09. Finally, non-completers had 3 or more birth complications more often than completers (6.2% against 1.9%), χ^2^(3) = 10.02, *p* = 0.018, V = 0.09. Among fathers, non-completers more often had no university degree than completers (60.4% against 42.6%), χ^2^(1) = 12.17, *p* < 0.001, ϕ = 0.12.

### Regression analyses

#### The associations of MOD and birth experience

**Fig. 3:**
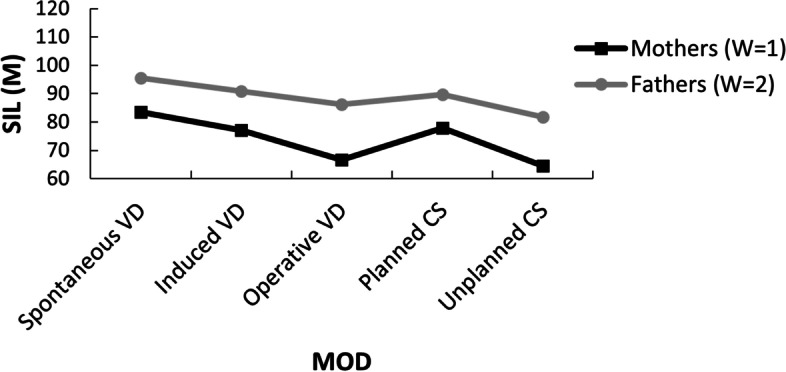
Simple slopes of the relative conditional effects ${\theta }_{MOD\to M}$

Among women, all categories of the MOD, i.e., induced VD, operative VD, planned CS, and unplanned CS predicted a more negative birth experience respectively compared to spontaneous VD. These results are displayed in Table 3 and graphically visualised with simple slopes in Fig. 3. Further, mothers experienced operative VD approximately as negative as unplanned CS. Among fathers, a similar pattern was found. All categories of the MOD, i.e., induced VD, operative VD, planned CS, and unplanned CS predicted a more negative birth experience respectively compared to spontaneous VD. On average, fathers also reported the most negative birth experiences when their baby was delivered via operative VD or unplanned CS. Still, overall fathers reported more positive birth experiences and the mean differences between MOD categories differed on a smaller range compared to mothers (see Table 3, Fig. 3). Only the effect of operative VD on birth experience was moderated by parental sex (*b* = 7.609, *p* < 0.05, see Table 4).

**Table 3:** Relative conditional effects of the MOD (X) on birth experience (M)^a^

	effects	*SE* ^b^	*p*	95% CI
$\left.{\theta }_{Induced VD\to M}\right|(W=1)$	-6.420^***^	1.532	< .001	[-9.425; -3.416]
$\left.{\theta }_{Induced VD\to M}\right|(W=2)$	-4.657^**^	1.650	.005	[-7.892; -1.421]
$\left.{\theta }_{Operative VD\to M}\right|\left(W=1\right)$	-16.810^***^	2.257	< .001	[-21.236; -12.383]
$\left.{\theta }_{Operative VD\to M}\right|(W=2)$	-9.201^***^	2.454	< .001	[-14.014; -4.388]
$\left.{\theta }_{Planned CS\to M}\right|\left(W=1\right)$	-5.592^*^	2.716	.040	[-10.919; -0.265]
$\left.{\theta }_{Planned CS\to M}\right|(W=2)$	-5.805^*^	2.274	.011	[-10.265; -1.345]
$\left.{\theta }_{Unplanned CS\to M}\right|\left(W=1\right)$	-18.944^***^	2.323	< .001	[-23.501; -14.386]
$\left.{\theta }_{Unplanned CS\to M}\right|(W=2)$	-13.765^***^	2.466	< .001	[-18.601; -8.929]

*MOD* mode of delivery, *VD* vaginal delivery, *CS* cesarean section, *CI* confidence interval; W = 1 = mothers; W = 2 = fathers; *p < .05; **p < .01; ***p < .001

^a^Controlling for parity, academic degree, prenatal symptoms of depression, birth complications, timing of holding the baby for the first time

^b^HC4 heteroskedasticity consistent standard error

**Table 4:** Regression coefficients for analysis 1^a^

Outcome										
Birth experience (M)						Parent-infant-bonding at T2 (Y)				
Predictors		*b*	*SE* ^b^	*p*	95% CI^c^		*b*	*SE* ^b^	*p*	95% CI^c^
Constant	i_M_	78.099	1.965	< .001	[74.245; 81.954]	i_Y_	17.010	4.275	< .001	[8.625; 25.396]
Induced VD	a_1_	-8.184^*^	3.471	.019	[-14.992; -1.376]	c’_1_	-2.398	1.815	.187	[-5.958; 1.162]
Operative VD	a_2_	-24.418^***^	5.111	< .001	[-34.443; -14.393]	c’_2_	-4.030	2.601	.122	[-9.131; 1.071]
Planned CS	a_3_	-5.379	5.908	.363	[-16.967; 6.210]	c’_3_	-4.771^*^	2.402	.047	[-9.481; -0.060]
Unplanned CS	a_4_	-24.122^***^	5.278	< .001	[-34.478; -13.766]	c’_4_	-3.607	2.470	.144	[-8.451; 1.236]
Sex	a_5_	11.964^***^	1.158	< .001	[9.693; 14.235]	c’_5_	4.729	3.095	.127	[-1.342; 10.800]
Induced VD x Sex	a_6_	1.764	2.245	.432	[-2.640; 6.167]	c’_6_	0.850	1.155	.462	[-1.415; 3.115]
Operative VD x Sex	a_7_	7.609^*^	3.314	.022	[1.109; 14.108]	c’_7_	2.069	1.819	.256	[-1.500; 5.638]
Planned CS x Sex	a_8_	-0.213	3.558	.952	[-7.192; 6.766]	c’_8_	1.993	1.668	.233	[-1.280; 5.265]
Unplanned CS x Sex	a_9_	5.178	3.403	.128	[-1.496; 11.853]	c’_9_	0.321	1.653	.846	[-2.931; 3.562]
SIL	-	-	-	-	-	b_1_	-0.115^**^	0.044	.009	[-0.201; -0.029]
SIL x Sex	-	-	-	-	-	b_2_	-0.030	0.032	.337	[-0.092; 0.032]
*R*^2^ = .257						*R*^2^ = .147				
*F*(14, 1609) = 41.946, *p* < .001						*F*(16, 1607) = 74.328, *p* < .001				

*VD* vaginal delivery, *CS* cesarean section, *SIL* Salmon’s Item List, *T2* around 8 weeks after the expected birth date

^a^Controlling for parity, academic degree, prenatal symptoms of depression, birth complications, timing of holding the baby for the first time

^b^HC4 heteroskedasticity consistent standard error

^c^Percentile bootstrap intervals based on 5,000 iterations

^*^p < .05; **p < .01; ***p < .001

#### The association of birth experience and parent-infant-bonding

A more positive birth experience predicted lower PBQ scores at T2 in mothers and fathers, indicating stronger parent-infant-bonding (*b* = -0.115, *p* < 0.01, see Table 4). This effect was not moderated by parental sex (*b* = -0.030, *p* > 0.05). As shown in Table 5, birth experience was not a statistically significant predictor of parent-infant-bonding at T3 anymore (*b* = -0.032, *p* > 0.05).

**Table 5:** Regression coefficients for analysis 2^a^

	Outcome Parent-infant-bonding at T3 (Y)				
Predictors		*b*	*SE* ^b^	*p*	95% CI^c^
Constant	i_Y_	10.941	4.095	.008	[2.910; 18.973]
Induced VD	c’_1_	0.323	1.913	.866	[-3.428; 4.075]
Operative VD	c’_2_	-2.484	2.876	.388	[-8.126; 3.157]
Planned CS	c’_3_	-5.137^*^	2.488	.039	[-10.016; -0.258]
Unplanned CS	c’_4_	-5.083	2.660	.056	[-10.301; 0.135]
Sex	c’_5_	6.957^*^	2.999	.021	[1.074; 12.839]
Induced VD x Sex	c’_6_	-0.922	1.237	.456	[-3.348; 1.504]
Operative VD x Sex	c’_7_	0.496	1.846	.788	[-3.125; 4.117]
Planned CS x Sex	c’_8_	2.156	1.700	.205	[-1.178; 5.489]
Unplanned CS x Sex	c’_9_	1.339	1.754	.445	[-2.102; 4.780]
SIL	b_1_	-0.032	0.043	.457	[-0.115; 0.052]
SIL x Sex	b_2_	-0.066^*^	0.031	.032	[-0.126; -0.006]
*R*^2^ = .097					
*F*(16, 1595) = 82.129, *p* < .001					

*VD* vaginal delivery, *CS* cesarean section, *SIL* Salmon’s Item List, *T3* around 14 months after the actual birth

^a^Controlling for parity, academic degree, prenatal symptoms of depression, birth complications, timing of holding the baby for the first time

^b^HC4 heteroskedasticity consistent standard error

^c^Percentile bootstrap intervals based on 5,000 iterations

^*^p < .05; **p < .01; ***p < .001

**Fig. 4:**
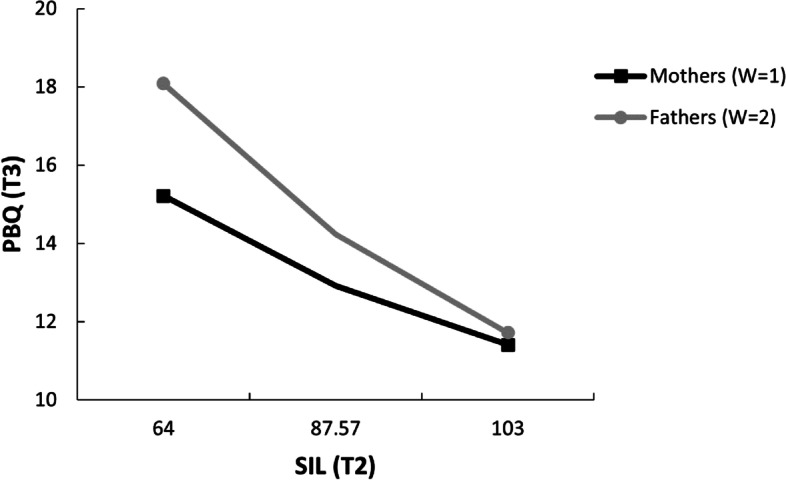
The interaction effect of birth experience and parental sex*. Note.* The 16^th^, 50^th^, and 84^th^ percentiles of the SIL distribution are displayed on the x-axis. SIL = Salmon's Item List; PBQ = Postpartum Bonding Questionnaire; T2 = around 8 weeks after the expected birth date; T3 = around 14 months after the actual birth

However, parental sex was a statistically significant predictor (*b* = 6.957, *p* < 0.05) of parent-infant-bonding at T3 and there was a statistically significant interaction effect between the birth experience and parental sex (*b* = -0.066, *p* < 0.05). When parents reported a more negative birth experience (lower SIL scores), fathers reported more bonding difficulties than mothers (higher PBQ scores). This is illustrated in Fig. 4. As shown, the more positive the birth experience becomes, the more the difference in the PBQ scores between mothers and fathers decreases. This suggests that a more negative birth experience might have a greater adverse impact on parent-infant-bonding at T3 for fathers than for mothers.

#### The relative conditional direct and indirect effects of the MOD on parent-infant-bonding at T2

Next, we explored whether the MOD predicted parent-infant-bonding at T2, both directly and indirectly (through the birth experience). The results are presented in Table 6. For mothers, the relative conditional direct effect of the MOD on parent-infant-bonding at T2 was statistically significant for planned CS and unplanned CS. That is, mothers who delivered their baby via planned CS or unplanned CS reported stronger parent-infant-bonding at T2 (lower PBQ scores) compared to mothers delivering their baby via spontaneous VD. For fathers, only unplanned CS had a statistically significant relative conditional direct effect on parent-infant-bonding at T2. That is, fathers whose baby was delivered via unplanned CS reported stronger parent-infant-bonding at T2 (lower PBQ scores) compared to fathers whose baby was delivered via spontaneous VD.

**Table 6:** Relative conditional effects of the MOD (X) on parent-infant-bonding at T2 (Y)^a^

Direct effect X➔ Y					Indirect effect X ➔ M ➔ Y			
	effects	*SE* ^b^	*p*	95% CI^c^		effects	*SE* ^d^	95% CI^c^
$\left.{\theta }_{Induced VD\to Y}\right|\left(W=1\right)$ $\left.{\theta }_{Induced VD\to Y}\right|(W=2)$	-1.548 -0.698	0.811 0.828	.056 .340	[-3.137; 0.042] [-2.322; 0.927]	${\theta }_{Induced VD\to M}\left.{\theta }_{M\to Y}\right|(W=1)$ ${\theta }_{Induced VD\to M}\left.{\theta }_{M\to Y}\right|(W=2)$	0.932 0.817	0.245 0.307	[0.480; 1.429] [0.246; 1.469]
$\left.{\theta }_{Operative VD\to Y}\right|\left(W=1\right)$ $\left.{\theta }_{Operative VD\to Y}\right|(W=2)$	-1.961 0.108	1.071 1.466	.067 .941	[-4.062; 0.140] [-2.768; 2.984]	${\theta }_{Operative VD\to M}\left.{\theta }_{M\to Y}\right|(W=1)$ ${\theta }_{Operative VD\to M}\left.{\theta }_{M\to Y}\right|(W=2)$	2.441 1.614	0.450 0.493	[1.638; 3.381] [0.734; 2.643]
$\left.{\theta }_{Planned CS\to Y}\right|\left(W=1\right)$ $\left.{\theta }_{Planned CS\to Y}\right|(W=2)$	-2.778^**^ -0.786	0.987 1.322	.005 .553	[-4.715; -0.842] [-3.379; 1.807]	${\theta }_{Planned CS\to M}\left.{\theta }_{M\to Y}\right|(W=1)$ ${\theta }_{Planned CS\to M}\left.{\theta }_{M\to Y}\right|(W=2)$	0.812 1.018	0.403 0.433	[0.036; 1.631] [0.229; 1.929]
$\left.{\theta }_{Unplanned CS\to Y}\right|\left(W=1\right)$ $\left.{\theta }_{Unplanned CS\to Y}\right|(W=2)$	-3.287^**^ -2.966^*^	1.048 1.250	.002 .018	[-5.342; -1.233] [-5.417; -0.515]	${\theta }_{Unplanned CS\to M}\left.{\theta }_{M\to Y}\right|(W=1)$ ${\theta }_{Unplanned CS\to M}\left.{\theta }_{M\to Y}\right|(W=2)$	2.751 2.415	0.480 0.549	[1.846; 3.743] [1.427; 3.569]

*MOD* mode of delivery, *VD* vaginal delivery, *CS* cesarean section, *CI* confidence interval, W = 1 = mothers, W = 2 = fathers

^a^Controlling for parity, academic degree, prenatal symptoms of depression, birth complications, timing of holding the baby for the first time; T2 = around 8 weeks after the expected birth date

^b^HC4 heteroskedasticity consistent standard error

^c^Percentile bootstrap intervals based on 5,000 iterations

^d^Bootstrapped standard error based on 5,000 iterations

^*^p < .05; **p < .01

In contrast to the relative conditional direct effects, the relative conditional indirect effects consider the effect of MOD on parent-infant-bonding through the birth experience. Among mothers, the birth experience mediated the association between induced VD, as well as planned CS and parent-infant-bonding at T2. Among fathers, the birth experience mediated the association between induced VD, operative VD, as well as planned CS and parent-infant-bonding at T2.

That is, an induced VD and planned CS, mediated by birth experience, seemed to be associated with weaker parent-infant-bonding at T2 in mothers (higher PBQ scores) compared to spontaneous VD. The same pattern was found among fathers, with the addition of the category operative VD. That is, an induced VD, an operative VD, and a planned CS, mediated by birth experience, seemed to be associated with weaker parent-infant-bonding at T2 in fathers (higher PBQ scores) compared to spontaneous VD.

#### The relative conditional direct and indirect effects of the MOD on parent-infant-bonding at T3

The relative conditional direct and indirect effects at T3 are reported in Table 7. For mothers, the direct effect of the MOD on parent-infant-bonding at T3 was statistically significant for planned CS and unplanned CS. This corresponds to the effects that were found at T2, thus, mothers who delivered their baby via planned CS or unplanned CS reported stronger parent-infant-bonding at T3 (lower PBQ scores) compared to mothers delivering their baby via spontaneous VD. For fathers on the other hand, no category of the MOD had a direct effect on parent-infant-bonding at T3. Regarding the indirect effect of the MOD on parent-infant-bonding at T3 through the birth experience, mothers and fathers showed a similar pattern.

**Table 7:** Relative conditional effects of the MOD (X) on parent-infant-bonding at T3 (Y)^a^

Direct effect X ➔ Y					Indirect effect X ➔ M ➔ Y			
	effects	*SE* ^b^	*p*	95% CI^d^		effects	*SE* ^c^	95% CI^d^
$\left.{\theta }_{Induced VD\to Y}\right|\left(W=1\right)$ $\left.{\theta }_{Induced VD\to Y}\right|\left(W=2\right)$	-0.599 -1.521	0.839 0.900	.476 .091	[-2.244; 1.047] [-3.287; 0.245]	${\theta }_{Induced VD\to M}\left.{\theta }_{M\to Y}\right|(W=1)$ ${\theta }_{Induced VD\to M}\left.{\theta }_{M\to Y}\right|(W=2)$	0.645 0.802	0.187 0.301	[0.314; 1.048] [0.257; 1.451]
$\left.{\theta }_{Operative VD\to Y}\right|\left(W=1\right)$ $\left.{\theta }_{Operative VD\to Y}\right|\left(W=2\right)$	-1.989 -1.492	1.273 1.335	.119 .264	[-4.485; 0.508] [-4.111; 1.126]	${\theta }_{Operative VD\to M}\left.{\theta }_{M\to Y}\right|(W=1)$ ${\theta }_{Operative VD\to M}\left.{\theta }_{M\to Y}\right|(W=2)$	1.633 1.482	0.360 0.442	[0.989; 2.390] [0.677; 2.406]
$\left.{\theta }_{Planned CS\to Y}\right|\left(W=1\right)$ $\left.{\theta }_{Planned CS\to Y}\right|\left(W=2\right)$	-2.981^**^ -0.826	1.053 1.344	.005 .539	[-5.046; -0.916] [-3.461; 1.810]	${\theta }_{Planned CS\to M}\left.{\theta }_{M\to Y}\right|(W=1)$ ${\theta }_{Planned CS\to M}\left.{\theta }_{M\to Y}\right|(W=2)$	0.533 0.878	0.280 0.409	[0.012; 1.111] [0.131; 1.758]
$\left.{\theta }_{Unplanned CS\to Y}\right|\left(W=1\right)$ $\left.{\theta }_{Unplanned CS\to Y}\right|\left(W=2\right)$	-3.744^***^ -2.404	1.150 1.313	.001 .067	[-6.000; -1.488] [-4.980; 0.171]	${\theta }_{Unplanned CS\to M}\left.{\theta }_{M\to Y}\right|(W=1)$ ${\theta }_{Unplanned CS\to M}\left.{\theta }_{M\to Y}\right|(W=2)$	1.857 2.256	0.401 0.530	[1.131; 2.730] [1.319; 3.469]

*MOD* mode of delivery, *VD* vaginal delivery, *CS* cesarean section, *CI* confidence interval, W = 1 = mothers; W = 2 = fathers; T3 = around 14 months after the actual birth

^a^Controlling for parity, academic degree, prenatal symptoms of depression, birth complications, timing of holding the baby for the first time

^b^HC4 heteroskedasticity consistent standard error

^c^Bootstrapped standard error based on 5,000 iterations

^d^Percentile bootstrap intervals based on 5,000 iterations

**p < .01; ***p < .001

The birth experience mediated the association between induced VD, operative VD, as well as planned CS and parent-infant-bonding at T3 in both mothers and fathers. That is, an induced VD, operative VD, and planned CS, mediated by birth experience, seemed to be associated with weaker parent-infant-bonding at T3 in both parents (higher PBQ scores) compared to spontaneous VD.

#### Test for moderation of the relative conditional indirect effects

Lastly, the indices of a moderated mediation [80] in both models with parent-infant-bonding at T2 (analysis 1) and T3 (analysis 2) were examined with regard to putative differences in indirect effects of mothers and fathers (i.e., a moderated mediation). The results indicated no differences.

## Discussion

The aim of this study was to prospectively investigate the role of MOD and birth experience for parent-infant-bonding in both mothers and fathers. All categories of the MOD predicted more negative birth experiences in mothers and fathers compared to spontaneous VD. A more positive birth experience predicted stronger parent-infant-bonding at 8 weeks postpartum, but not at 14 months postpartum. In mothers who delivered their baby via planned CS or unplanned CS parent-infant-bonding was stronger at 8 weeks and 14 months postpartum compared to spontaneous VD. In fathers whose baby was delivered via unplanned CS parent-infant-bonding was stronger at 8 weeks postpartum compared to spontaneous VD. However, in fathers no associations between MOD and parent-infant-bonding were found at 14 months postpartum.

The birth experience mediated the association of induced VD, planned CS with mother-infant-bonding and of induced VD, operative VD, and planned CS with father-infant-bonding at 8 weeks postpartum. At 14 months postpartum, the birth experience mediated the association of induced VD, operative VD, and planned CS with parent-infant-bonding in both mothers and fathers.

### The associations of MOD and birth experience

Both mothers and fathers experienced the birth of their infant via induced VD, operative VD, planned CS, and unplanned CS more negative than parents, whose baby was delivered via spontaneous VD, indicating that the MOD is a relevant factor contributing to the self-reported birth experience of parents. These findings are contributing to previous research and, especially in fathers, extending the evidence to two more MODs, i.e., induced VD and planned CS. One factor in explaining these results could be the violation of expectations [83, 84], as at least most mothers wish for a spontaneous VD [16]. Further, Handelzalts et al. [85] emphasize the aspect of a planned delivery that seems to be associated with a more positive birth experience in mothers compared to unplanned deliveries or interventions. This might also contribute to the fact that a planned CS was not perceived as equally negative as an unplanned CS in both parents. While it is also a CS, it is a planned procedure that parents can prepare for and know what to expect. Also, Kjerulff et al. [86] showed that women undergoing a planned or unplanned CS were least likely to be proud of themselves right after childbirth, and especially women who delivered their baby via unplanned CS often felt disappointed or even like a failure. Therefore, these negative emotions around a more medicalized birth might contribute to an overall more negative birth experience in mothers. In fathers, there is evidence pointing out negative emotions in participating in childbirth such as feeling overwhelmed, helpless, excluded, and anxious during labour and birth [47, 87–89] that might be worth considering here. According to these studies, fathers often feel insecure about the occurrences during childbirth and the mother’s labor pain, not knowing what to do about it or how to help. Vallin et al. [90] also highlighted these emotions particularly in the light of a more complicated childbirth. Hence, it is conceivable that these emotions contribute to a more negative birth experience in fathers. Another important factor might be that birth preparation courses do not seem to sufficiently prepare fathers for childbirth and the potentially occurring complications [91]. However, the differences in birth experiences between the MODs were smaller in fathers than in mothers in our study. Therefore, the overall challenging situation of a more complicated childbirth, requiring the intervention of healthcare professionals, might be more important to fathers than the exact procedures and interventions linked to the different categories of the MOD. In support of this Johansson et al. [92] showed that in the situation of an impending CS, the father’s concern for the safety of mother and infant outweighed his interest in the MOD, he just wanted them to be safe. However, the father might also feel the mother’s disappointment with not delivering via spontaneous VD, which conceivably could have an impact on him. Overall, mothers reported more negative birth experiences compared to fathers, which is plausible due to various aspects, including e.g., the obstetric interventions being performed on them. Nevertheless, the father’s birth experiences should not be marginalized. Etheridge et al. [93] showed that facing a traumatic birth, fathers invalidated their own feelings by stating that since they had not experienced birth themselves, they had no grounds for justification.

### The association of birth experience and parent-infant-bonding

A more positive birth experience predicted stronger parent-infant-bonding in mothers and fathers at 8 weeks postpartum, but not at 14 months postpartum. One explanation for this finding might be the hormone oxytocin, which is released during labour and birth to regulate maternal stress and pain as well as stimulate positive feelings such as joy and happiness [94]. Oxytocin plays a crucial role in the establishment and promotion of mother-infant-bonding [95]. Therefore, it seems plausible that higher oxytocin levels during a more positively experienced birth also affect the first interactions of the mother with her infant, resulting in a stronger bond at 8 weeks postpartum. However, these hormonal influences probably did not play a significant role anymore 14 months after birth. In fathers, oxytocin levels also seem to increase in the first months of fatherhood and facilitate caregiving behaviours [96]. In addition, Gettler et al. [97] showed that oxytocin levels were higher in fathers after holding the infant for the first time after childbirth compared to before. The authors also found interaction effects of testosterone in predicting father-infant-bonding. Therefore, hormonal changes might also be of importance in facilitating stronger parent-infant-bonding in fathers. However, these hormonal processes during childbirth and their impact on father-infant-bonding need to be further studied. Another explanation to this finding might be the positive mental state that parents are in when they experience birth as more positive, which might in turn facilitate stronger bonding. Indeed, since greater maternal well-being seems to be associated with stronger prepartum mother-infant-bonding [98], the same could be true for postpartum bonding, and taking a broader approach, for fathers as well. Another explanation could be that parent-infant-bonding develops gradually over the first few weeks and months after birth, and factors other than the immediate birth experience influence parent-infant-bonding more strongly 14 months postpartum. For instance, Takács et al. [99] showed that the infant’s temperament 6 weeks postpartum predicted mother-infant-bonding 9 months postpartum.

Also, when parents experienced a more negative birth, fathers reported weaker parent-infant-bonding at 14 months postpartum than mothers. One explanation might be that fathers might not have talked much about and in turn processed their negative birth experience, thereby giving this event more space influencing father-infant-bonding. In support of this, a recent narrative review by Masoumi [100] showed that fathers are more inclined to hide their tokophobia, which has a negative impact on coping with it. Considering that fathers might question the justification of their feelings in this regard anyway as Etheridge et al. [93] showed and embarrassment seems to be a key factor of why men do not seek for help [101], this might have also played a role here. Another finding suggesting that a more negative birth experience might be more meaningful regarding the impact on parent-infant-bonding for fathers was that the birth experience mediated the association between weaker parent-infant-bonding in more categories of the MOD in fathers than in mothers. However, this remains a subject of debate and should be further studied, particularly examining the additional support that fathers might need when experiencing the birth as more negative.

### The associations of MOD and parent-infant-bonding (relative conditional direct effects)

Mothers who delivered their baby via planned CS or unplanned CS reported stronger parent-infant-bonding at 8 weeks and 14 months postpartum compared to mothers delivering via spontaneous VD. In fathers, only the delivery of the infant via unplanned CS was associated with stronger parent-infant-bonding at 8 weeks postpartum compared to the delivery via spontaneous VD. At 14 months postpartum, no category of the MOD had a direct effect on parent-infant-bonding among fathers. Apparently, these findings are contradictory to previous research, finding either no association between the MOD and mother-infant-bonding [26–29] or indeed weaker mother-infant-bonding in mothers giving birth by CS compared to spontaneous VD [30, 31]. An explanation to our findings might be compensatory behaviors that parents, whose baby was delivered via unplanned CS, engage in to mitigate potential negative impacts for the infant. A mechanism previously discussed over the fact that mothers of preterm infants seem to report stronger mother-infant-bonding compared to mothers of full-term infants [70, 102], might support this hypothesis, as the situations are similar. The authors propose the explanation of the *compensatory care theory*, linking more parental care to parents with preterm babies, aiming to compensate for adverse consequences for the preterm infant at higher risk [103]. This approach might also apply here, with a CS being a situation of high risk for both mother and infant that might lead to compensatory behaviors of parents to facilitate stronger bonding. As can be seen in the size of the effects (Tables 6 and 7), the direct effect was indeed the largest for unplanned CS in mothers at both measurement points and in fathers at 8 weeks postpartum, which might also support this hypothesis.

Another important aspect potentially accounting for the conflicting evidence might be the birth experience, which had not been considered in prior studies that investigated the direct association between MOD and parent-infant-bonding. Due to our mediation model, the relative conditional direct effects are controlled for the birth experience in this study and therefore might be more reliable for assessing the direct effect of MOD on parent-infant-bonding. As the relative conditional direct and indirect effects indicate, controlling for or mediating by birth experience may make a difference in presence or direction of the associations found between MOD and parent-infant-bonding.

### The mediating role of birth experience (relative conditional indirect effects)

Among mothers, the birth experience mediated the association between induced VD as well as planned CS and weaker parent-infant-bonding at 8 weeks postpartum. Among fathers, the same associations were found at that measurement point with the addition of operative VD. At 14 months postpartum, the birth experience mediated the association between induced VD, operative VD, and planned CS and weaker parent-infant-bonding in both parents. Thus, considering the mediation effects, i.e., the effect of the MOD on parent-infant-bonding mediated by the birth experience, our results are in line with previous studies, linking especially a CS with weaker mother-infant-bonding [30, 31]. Also, induced VD and operative VD only had an indirect effect on parent-infant-bonding through the birth experience. Interestingly, planned CS had both a relative conditional direct and indirect effect in mothers, unfolding reversed effects on parent-infant-bonding. This might seem contradictory at first glance, when in fact it is an example of the Yule-Simpson Paradox [104, 105]. A reversal of the effects occurs when the birth experience is entered into the association as the mediator. Among women giving birth via planned CS, there were some women who reported a more positive birth experience and stronger parent-infant-bonding compared to mothers giving birth by spontaneous VD. Some of them even explicitly requested a CS due to personal reasons. Considering that a more *positive* birth experience predicted *stronger* parent-infant-bonding, this is a logical result, contributing to the relative conditional direct effect that we found. Among those delivering via planned CS *and* experiencing the birth as more *negative* compared to spontaneous VD, the reported bonding seemed to be *weaker*, which explains the relative conditional indirect effect that we found. Therefore, it seems to be the more negative birth experience that accounts for the reverse effects between associations with and without the mediation effect. Although, looking at the bootstrapped 95% CI, it is important to notice that the relative conditional indirect effects of planned CS in mothers at T2 and T3 were close to being statistically insignificant, whereas the relative conditional direct effects were statistically significant at p < 0.01. Further studies should address whether the indirect effects can be replicated or whether the direct effects might be of greater importance.

Nonetheless, in at least some cases, the birth experience operated as a mediator of the association between MOD and parent-infant-bonding and even reversed the association of planned CS and parent-infant-bonding in mothers. Hence, our study’s results suggest that investigating the association between MOD and parent-infant-bonding should not be conducted without considering the birth experience.

### Strengths and limitations

Several strengths of the present study can be highlighted. Data of the large prospective cohort study DREAM were used, thereby enabling the investigation of the longitudinal associations of the MOD, birth experience, and parent-infant-bonding, while controlling for relevant confounding variables. Besides, this was the first study to include the father in the investigation of the association between MOD and parent-infant-bonding. In addition, a novelty of this study was the examination of the birth experience as a mediator in both mothers and fathers. Hence, this study extends the existing literature in this field. Moreover, our results provide a possible clarification to the heterogeneous body of literature, as the birth experience was shown to be a mediator in explaining the association between some categories of the MOD and weaker parent-infant-bonding in our study. Another strength of our study was the large sample size and use of validated instruments of high psychometric quality. The PBQ was found to be the most studied, evidence based instrument with high psychometric qualities for measuring postpartum bonding in a recent systematic review by Wittkowski et al. [106]. Besides, we contrasted four different categories of MOD against a spontaneous VD, while other studies often examined MOD in a less detailed manner.

However, some limitations of this study should be noted. For one, self-selection bias known in epidemiologic studies [107–109] did also apply here, with more participants holding a university degree than not. Compared to the general population of Dresden, our sample was thus rather highly educated [110]. Additionally, the dropout analyses showed that in mothers, non-completers reported higher prepartum symptoms of depression, were more often unemployed, less educated, and experienced more birth complications and more often a delivery via unplanned CS. Maternal non-completers can therefore be considered a more vulnerable group than completers, which could have been a reason for dropping out of our study. Also, our participants reported overall strong bonding with their infants and there was a relatively small variance in the reported scores. Therefore, the results of our rather homogenous sample of first-time parents should not be generalized but encourage research in more heterogeneous samples. One limitation to our study that needs to be considered is the lack of data regarding the prepartum parent-infant-bonding as prepartum mother-infant-bonding seems to be positively associated with the postpartum bond [56, 111]. Another limitation is that we did not collect data on, and thus did not consider, the administration of epidural analgesia during birth that seems to be associated with mother-infant-bonding [112, 113].

Additionally, we only considered prenatal symptoms of depression as a confounding variable, although also postpartum depressive symptoms seem to be linked with weaker mother-infant-bonding [114, 115]. Yet, Paulson et al. [116] showed that symptoms of depression remained stable from pregnancy until 6 months postpartum in both mothers and fathers. Another minor limitation may be that we combined a CS due to personal or medical reasons into one category (planned CS) prior to analyses. Future studies with a larger sample size of women or parents choosing a CS due to personal reasons should be further investigating this MOD regarding birth experience and parent-infant-bonding.

### Research and practical implications

Further research is necessary investigating the mediating role of the birth experience for the association between MOD and parent-infant-bonding in more detail and attempting to replicate our findings. In particular, the association between an unplanned CS and parent-infant-bonding should be further studied, examining mechanisms like the compensatory care theory [103]. As we found no mediating effect for unplanned CS, it should be studied if the birth experience might not be as influential regarding unplanned CS compared to other MODs and which specific protective factors might be of importance. Also, future studies similar to ours but in clinical samples, potentially investigating bonding *disorders* seem very promising. In addition, studies using observational procedures to assess infant’s attachment to their parents and interaction between infants and parents would complement the parent’s perspective and provide a more comprehensive picture of the parent-infant-relationship. Generally, fathers should more often be included in these studies, as mothers and fathers might have differential needs in e.g., making a more positive birth experience possible. As we emphasized the importance of a positive birth experience, conditions in hospital care under which obstetricians and midwives work, should be improved [117, 118], thus providing further support for enabling a positive birth experience. Moderate to high levels of burnout syndrome in nurses [119], midwives [120], and obstetricians [121] underline this problem and the need for change in working conditions, as it seems to negatively affect the provided quality of care [122]. For fathers dealing with negative emotions during childbirth, it seems especially important how the medical staff interacts with and supports them [44, 47, 48]. In mothers, the feelings of disappointment and failure giving birth by CS [86] should be gently addressed or, better yet, prevented. Tully et al. [123] refer to the *social stigma* that delivering via CS (planned or unplanned) seems to be attached to, in terms of avoiding the strenuous process of childbirth, seemingly choosing the somewhat simpler way of delivering. This picture does not do justice to the delivery via CS, indeed it neglects the clinical and psychological indications for a planned or unplanned CS [124–126]. Socially destigmatizing a CS and providing more information about it especially before birth in e.g., birth preparation courses could be helpful not to let shameful feelings arise in mothers in the first place. After a CS midwives and doulas should emotionally support mothers, thereby potentially buffering against a negative birth experience and shame or judgment that mothers might feel exposed to or be afraid of.

## Conclusion

We pioneered to show the mediating role of the birth experience in both mothers and fathers for some categories of the MOD and weaker parent-infant-bonding at both 8 weeks and 14 months postpartum. This underscores the importance of a positive birth experience for both parents, which should receive more attention in both obstetrical care and in research by examining its influence in several relevant associations for parents and their infants. Hence, our study suggests that the impact of the MOD on parent-infant-bonding should not be investigated without the consideration of the birth experience. Further studies should investigate whether our results regarding the impact of an unplanned CS on parent-infant-bonding can be replicated. What is more, our study highlights that fathers might have differential needs than mothers that should be targeted in birth preparation courses and hospital care. With e.g., improving the hospital conditions for obstetricians and midwifes and addressing the father more in birth preparation courses, enabling a more positive birth experience for both parents and stronger parent-infant-bonding might be facilitated. As especially stronger parent-infant-bonding is shown to have positive impacts on the infant’s development [127, 128], this would be beneficial for the whole family system, including both parents and the infant.

## Data Availability

The dataset analyzed during the current study is not publicly available due to legal and ethical constraints as the study’s informed consent did not include public sharing of participant data. The dataset is available from the corresponding author on reasonable request.

## Abbreviations

MOD: Mode of delivery
VD: Vaginal delivery
CS: Cesarean section
DREAM: Dresden Study on Parenting, Work, and Mental Health (“DResdner Studie zu Elternschaft, Arbeit und Mentaler Gesundheit”)
T1: Measurement point during pregnancy
T2: Measurement point at 8 weeks after the expected birth date
T3: Measurement point at 14 months after the actual birth
SIL: Salmon’s Item List
PBQ: Postpartum Bonding Questionnaire
